# Flavonoid Glycosides from Endemic Bulgarian *Astragalus aitosensis* (Ivanisch.)

**DOI:** 10.3390/molecules24071419

**Published:** 2019-04-11

**Authors:** Hristo Vasilev, Samir Ross, Karel Šmejkal, Petr Maršík, Dagmar Jankovská, Jaroslav Havlík, Ondřej Veselý

**Affiliations:** 1 Department of Pharmacognosy, Faculty of Pharmacy, Medical University–Sofia, 2 Dunav str., 1000 Sofia, Bulgaria; christo.vasilev@yahoo.com; 2 National Center for Natural Products Research, School of Pharmacy, University of Mississippi, Oxford, MS 38677, USA; sross@olemiss.edu; 3 Department of Natural Drugs, Faculty of Pharmacy, University of Veterinary and Pharmaceutical Sciences Brno, 61242 Brno, Czech Republic; 4 Department of Food Science, Czech University of Life Sciences Prague, Kamycka 129, 165 00 Prague 6–Suchdol, Czech Republic; marsik@af.czu.cz (P.M.); havlik@af.czu.cz (J.H.); veselyo@af.czu.cz (O.V.); 5 Institute of Experimental Botany, Czech Academy of Sciences, Rozvojová 263, 16502 Prague, Czech Republic

**Keywords:** *Astragalus aitosensis*, flavonoid, glycoside

## Abstract

*Astragalus* is a very interesting plant genus, well-known for its content of flavonoids, triterpenes and polysaccharides. Its secondary metabolites are described as biologically active compounds showing several activities, e.g., immunomodulating, antibacterial, antiviral and hepatoprotective. This inspired us to analyze the Bulgarian endemic *A. aitosensis* (Ivanisch.) to obtain deeper information about its phenolic components. We used extensive chromatographic separation of *A. aitosensis* extract to obtain seven phenolic compounds (**1**–**7**), which were identified using combined LC-MS and NMR spectral studies. The 1D and 2D NMR analyses and HR-MS allowed us to resolve the structures of known compounds **5**–**7** as isorhamnetin-3-*O*-robinobioside, isorhamnetin-3-*O*-(2,6-di-*O*-α-rhamno-pyranosyl-β-galactopyranoside), and alangiflavoside, respectively, and further comparison of these spectral data with available literature helped us with structural analysis of newly described flavonoid glycosides **1**–**4**. These were described in plant source for the first time.

## 1. Introduction

Genus *Astragalus* comprises from more than 2500 species, which makes it the largest genus in the family Fabaceae. *Astragalus* species are cosmopolitans, widely distributed in dry and semi-dry regions, mainly in the temperate regions of the Northern hemisphere [1]. Certain sources assign genus *Astragalus* as the largest genus of flowering plants [2]. About 133 species are distributed in Europe [3], and 29 have been identified in Bulgaria [4,5]. Fourteen *Astragalus* species from the Bulgarian flora are protected by the Bulgarian Biodiversity Act and they are included in the Red List of Bulgaria, as is the species *A. aitosensis*, the focus in this investigation [4].

The object of our study—*A. aitosensis* (Ivanisch.) (syn. *Astracantha aitosensis* (Ivanisch.) Podlech, *Astracantha arnacantha* (M. Bieb.) Podlech subsp. *aitosensis* (Ivanisch.) Réer & Podlech)—is a Bulgarian endemic plant and yet it is very scarcely studied for its phytochemical composition [6,7]. *A. aitosensis* is a low, spiny, tussock-forming shrub with strongly branched stems (30–50 cm in height) [8]. The plant grows in dry stony places (90–550 m alt) with neutral to alkaline soil. It is distributed only in the suburbs of the small Bulgarian town Aytos, which gives rise to its name.

From pharmacological point of view, *Astragalus* species are well-known and widely used as remedies in the traditional folk medicine of different countries, but only about 100 species from the genus are researched for their phytochemical composition and properties. Their activity described in the literature is a result of the presence of flavonoids, saponins, and polysaccharides [9,10], which endue these plants with immunomodulating, antibacterial, antiviral, hepatoprotective and other protective pharmacological effects [1,9,10].

*Astagalus* species show diverse flavonoid composition: flavons, flavonols, flavanons, flavanonols, chalcones, isoflavons, isoflavans and pterocarpans [1]. Flavonols—in their free and glycosidic forms—are the most common aglycons, particularly kaempferol, quercetin and methylquercetin (syn. isorhamnetin). Astragalin, rutoside, hyperoside and isoquercitrin are the most frequently found glycosidic forms [1,10]. Interesting recent research by Bulgarian scientists reports the presence of a rarely-met group of flavo-alkaloids [11] and heavily glycosylated tri- and tetra-flavonols [10].

The aim of this study was to isolate content compounds of the methanolic extract from aerial parts of *A. aitosensis* and preform their structural elucidation using ^1^H, ^13^C, COSY, HSQC, HMBC, NOESY, and TOCSY NMR experiments. HR-ESI-MS was used for additional confirmation of the structures revealed by NMR. We report here the isolation and structural elucidation of six isorhamnetin and one kaempferol glycosides **1**−**7**, four of which are new natural glycosides: three tetra- (**1**–**3**) and one triglycoside (**4**). The other three already known structures were determined for the first time in *A. aitosensis*: with two (**5**), three (**6**) and four (**7**) sugar units, respectively.

## 2. Results

### 2.1. Isolation of Compounds

**Figure 1:**
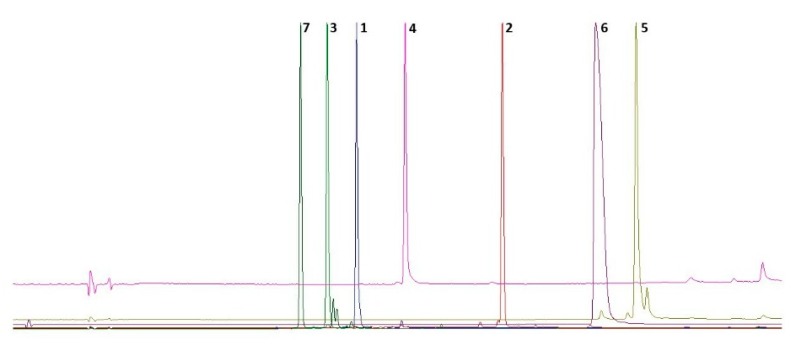
The exemplified overlay of chromatograms of compounds **1**–**7** at λ 254 nm.

The aerial parts of *A. aitosensis* were extracted with 80% methanol under reflux. The preliminary chromatographic analysis showed a bunch of signals of flavonoid compounds, with retention times predicting a high degree of glycosylation (Figure 1).

**Figure 2:**
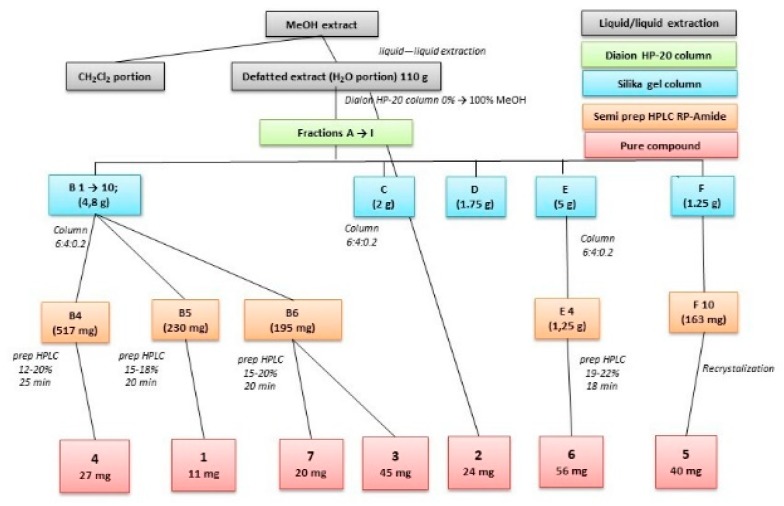
Simplified scheme of separation.

The crude extract was therefore defatted by liquid−liquid partitioning with chloroform and further fractionated via successive column chromatography with final step of semi-preparative HPLC purification of the isolated compounds (Figure 2). Their nature (UV spectral properties) and the behavior of these compounds during separation on reversed phase—the polar character of the compounds—gave us the idea of flavonoid glycosides with a high glycosylation pattern.

**Figure 3:**
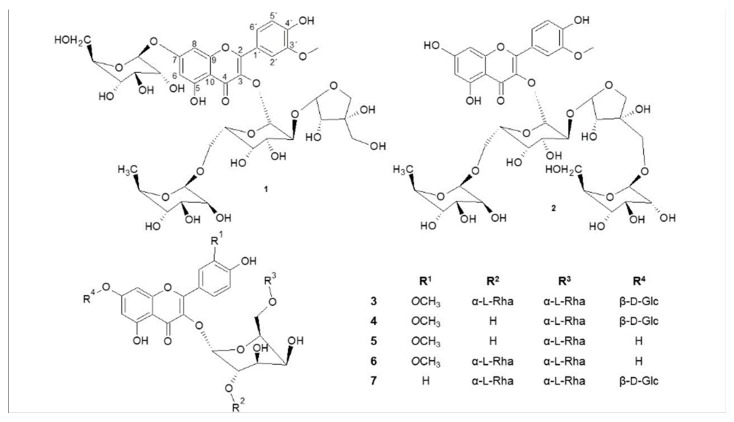
Structures of isolated compounds **1**–**7**.

Our separation procedures resulted in isolation of seven pure compounds (**1**–**7**) (Figure 3).

### 2.2. Structural Analysis

To the best of our knowledge, spectral data of four of the isolated compounds (**1**–**4**) did not correspond to the data of compounds previously published in the literature. The 1D and 2D-NMR analysis, HR-MS and comparison with literature allowed us to resolve the structures of compounds **5**–**7** as isorhamnetin-3-*O*-robinobioside [12], isorhamnetin-3-*O*-(2,6-di-*O*-α-rhamno-pyranosyl-β-galactopyranoside) [13,14], and alangiflavoside [15], respectively. The further comparison of these spectral data with available literature helped us with structural analysis of newly described flavonoid glycosides **1**–**4**. For a detailed description, please see Supplementary Materials, Figures S1–S77.

The aglycones for compounds **1**–**6** were determined based on HR-ESI-MS and NMR (^1^H, ^13^C, COSY, HSQC and HMBC) spectral analysis. ^1^H and ^13^C spectra are shown in Table 1. HMBC spectra showed the following significant correlations: proton at C-2′ was a doublet with *meta* coupling and, in the HMBC, it showed strong correlation to C-4′ and weak to C-3′. The proton at C-6′ was observed as doublet of doublet (*ortho* and *meta* coupling) and showed strong correlation in the HMBC to C-4′. The proton at C-5′ was a doublet with *ortho* coupling, displaying in the HMBC the strong correlation to C-3′ and weak to C-4′. The methoxy group showed correlation to C-3′, and, therefore, the position of the methoxy is at C-3′ and the aglycone of compounds **1**–**6** was finally identified as 3′-*O*-methylquercetin, syn. isorhamnetin [16].

Because of the identification procedure, we describe the elucidation of structures of known compounds prior to the new compounds. Detailed ^1^H and ^13^C chemical shifts for compound **5** are listed in Table 2 and Table 3, respectively. The LC-MS analysis showed single peak (*t*_R_ 7.95 min) chromatogram with a signal of a deprotonated molecule at *m/z* [M − H]^−^ 623.16205, which showed a good correlation with the calculated value of *m/z* 623.16176 for C_28_H_31_O_16_^-^ (Δ = 0.00029). The (+)HRMS supported the idea of this molecular formula; the glycosylation pattern was predicted to be the rhamnose and a hexose from MS/MS analysis of *m/z* [M + H]^+^ 625.1175 and observed fragments *m/z* [M + H − rhamnose]^+^ 479.1184, *m/z* [M + H − rhamnose-oxygen]^+^ 463.1213, and finally *m/z* 317.0645 showing the aglycone. The molecular formula C_28_H_32_O_16_ (calcd 624.54408) accorded with the NMR and further HR-ESI-MS spectral data, and the data of compound **5** corresponded to the reported data for isorhamnetin-3-*O*-robinobioside [12].

ESI^–^ HRAM spectrum of compound **6** showed presence of signal of deprotonated molecule *m/z* [M − H]^−^ 769.21948 (calcd for C_34_H_41_O_20_^-^ 769.219667 *m*/*z*; Δ = 0.00019). The MS/MS analysis (a minimal fragmentation using 20 eV) in a positive mode showed the presence of a protonated parental ion *m/z* [M + H]^+^ 787.2284 and fragments corresponding to loss of two rhamnose units and a hexose *m/z* 641.1708, 479.1180 and 317.0653. The molecular formula C_34_H_42_O_20_ (Mr 770.68528) is in accordance with the NMR and HR-ESI-MS spectral data and ^1^H and ^13^C chemical shifts for compound **6** are listed in Table 2 and Table 3, respectively. Compound **6** was therefore identified as isorhamnetin-3-*O*-(2,6-di-*O*-α-rhamno-pyranosyl-β-galactopyranoside) [13,14].

The molecular formula C_39_H_50_O_24_ (Mr 902.7999) is in accordance with the NMR and HR-ESI-MS spectral data, where the mass of deprotonated molecule was found at *m/z* [M − H]^−^ 901.2624 (calcd 901.2619; Δ = 0.0005). The detailed MS analysis of fragments observed in spectrum showed signal *m/z* [M + H]^+^ 903.2759, *m/z* [M + H − rhamnose]^+^ 757.2178, *m/z* [M + H − rhamnose − rhamnose]^+^ 611.1601, *m/z* [M + H − rhamnose − rhamnose − hexose]^+^ 449.1078, and aglycone *m/z* 287.0549, with pairs of fragments showing loos of water 18 *m/z* [M + H − rhamnose − H_2_O]^+^ 741.2245, *m/z* [M + H − rhamnose − rhamnose − H_2_O]^+^ 595.1660. The spectrum also showed fragments of sugars *m/z* 309.1186 (hexose-rhamnose). ^1^H and ^13^C chemical shifts for compound **7** are listed in Table 2 and Table 3 respectively. NMR data of compound **7** and mass spectral analysis results show a good accordance with the data already reported for alangiflavoside [15].

**Figure 4:**
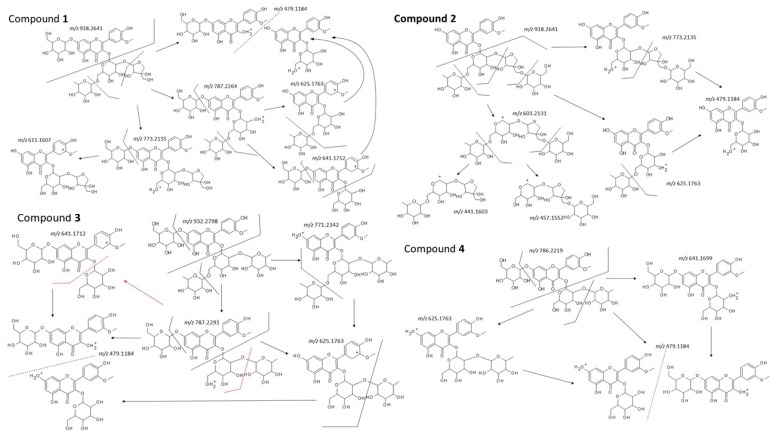
A fragmentation pattern of flavonoid glycosides **1**–**4** (shown *m/z* are calculated values according to molecular formulas of the ions).

Based on the previously described analysis of known compounds, we tried to identify the other isolated compounds, which showed differences from those previously described in the literature. Some of the fragmentation MS/MS results used for the identification of compounds **1**–**4** are depicted in Figure 4.

Compound **1** was isolated as a yellow amorphous powder. The UV spectra analysis showed a typical flavonoid course, with the retention time typical for glycosylated substances. The HR-ESI-MS in a negative mode displayed a molecular ion *m/z* [M − H]^−^ 917.2572 (calc. for [M − H]^−^ 917.256841), confirming the molecular formula C_39_H_50_O_25_ with calculated molecular mass 918.7993 Da. HRESIMS showed an adduct formation *m/z* [M + HCOO]^−^ 963.26269 (confirming the analyte), and a hexose (*m/z* 162.0235) loss leading to *m/z* 755.21208 (possible representing cleavage of glucose from 7-*O* position or galactose from 3-*O*- position of expected flavonol skeleton). Furthermore, the MS in a positive mode confirmed this loss showing precursor ion *m/z* [M + H]^+^ 919.2705 with product ions formed either by subsequent loses of a pentose (*m/z* 787.2264), a rhamnose (*m/z* 625.1750), and a hexose (479.1170) unit, or loss of rhamnose (*m/z* 773.2136) followed by hexose (*m/z* 611.1613) moieties (see Figure 4). After lining up interpretation of NMR spectra (Table 1) with MS analysis, the structure of compound **1** was elucidated to be the isorhamnetin substituted by four sugars. One of them is a 6-deoxyhexose (a methyl group as doublet), two are hexoses (a glucose, a galactose), and one is a pentose. DEPT spectrum of compound **1** showed four methylene groups. Altogether, two of them belong to each hexose (the glucose and galactose), respectively, and since a 6-deoxyhesose (expected rhamnose) does not have a methylene moiety, the two left CH_2_ moieties must belong to a pentose. Four anomeric protons are found in HMBC spectrum: δ_H_ 5.57 ppm, (1H, d, *J* = 7.84 Hz), δ_H_ 5.43 ppm (1H, d, *J* = 1.55 Hz), δ_H_ 4.50 ppm (1H, d, *J* = 1.56 Hz) and δ_H_ 5.06 ppm (1H, d, *J* = 7.53 Hz), corresponding to carbon atoms with δ_C_ 99.8 ppm, δ_C_ 109.2 ppm, δ_C_ 100.5 ppm and δ_C_ 100.1 ppm from the HSQC spectrum. After complete resonance assignments, analyses of coupling constants, intensities, interpretation of cross-peaks in the COSY spectrum, and ^13^C-NMR chemical shift values, one hexose moiety was identified as a β-glucosyl unit, the second as a β-galactosyl moiety, the 6-deoxysugar was identified to be the α-rhamnose, and the pentose was recognized as the β-apiose, which contains two of the above-mentioned methylene groups. ^13^C values of C-6 indicate that β-glucose residue is not connected to other sugar unit (δ_C_ 61.1 ppm), while the β-galactose moiety is connected at C-6 position (δ_C_ 66.0 ppm—shifted to a higher field). HMBC correlations allowed us to elucidate the precise structure of the sugar chains and the positions of their attachment to the aglycone. The anomeric proton (δ_H_ 5.06 ppm) of the β-glucose moiety showed a three-bond correlation to C-7 (δ_C_ 163.0 ppm) of the aglycone, while a HMBC correlation between the anomeric proton of the galactose (δ_H_ 5.57 ppm) and the carbon at δ_C_ 133.6 ppm indicated that the galactosyl unit is connected at C-3 towards the aglycone. The anomeric proton of α-rhamnose correlated to C-6 in the β-galactose (δ_C_ 66.0 ppm). The anomeric atom of the last pentose sugar (apiose) δ_H_ 5.43 ppm bonded to δ_C_ 109.2 ppm showed a HMBC correlation with C-2 of the β-galactose molecule (δ_C_ 75.1 ppm). The remaining two methylene groups (δ_C_ 74.2 ppm and δ_C_ 65.1 ppm) were recognized as carbons C-4 and C-5 in the apiosyl moiety. Hence, according to all interpreted spectra, we identified compound **1** as a new natural product named isorhamnetin-3-*O*-[β-d-apiofuranosyl-(1→2)-[α-l-rhamnopyranosyl-(1→6)]-β-d-galactopyranosyl]-7-*O*-β-d-glucopyranoside.

Compound **2** was obtained as a yellow amorphous powder. The spectral analysis showed data very similar to those observed for compound **1** (Table 1), and the only difference found was the presence of a β-glucosyl residue connected to the sugar chain, attached to the 3-*O* position (particularly C-5 of apiosyl residue); a connection to the 7-*O* position of the aglycone was not found. Further interpretation of HRESIMS supported this suggestion by observing a lacking of the fragment of a hexose loss (*m/z* 162.0235), which can be observed in spectra of all compounds possessing glucose attached at 7-*O* position (compounds **1**, **3**, **4** and **7**). The HR-ESI-MS in negative mode displayed a molecular ion *m/z* [M − H]^−^ 917.25696 (calculated for *m/z* [M − H]^−^ 917.256841), confirming molecular formula C_39_H_50_O_25_ with calculated molecular mass 918.7993 Da. Adduct formation [M + HCOO]^−^ (*m/z* 953.2336) and [M + Cl]^−^ (*m/z* 963.2621) was observed in a negative ESI mode. Furthermore, MS in a positive mode confirmed this by [M + H]^+^ with *m/z* 919.2709, and a series of corresponding losses of four sugar units at a minimal fragmentation of 20 eV: *m/z* 773.2136 as loss of rhamnosyl unit, *m/z* 625.1759 loss of rhamnose and hexose, and 479.1185 corresponding to isorhamnetin hexoside after loss of three sugar units of rhamnose, hexose and pentose. In addition, the aglycone signal *m/z* 317.0655 and the fragment ion *m/z* 603.2144, interpreted as the chain of all sugar moieties with corresponding fragments *m/z* 441.1610 and *m/z* 457.1552 after losses of terminal hexose and rhamnose, respectively, were detected. According to differences in chemical shifts in NMR spectral data (compared with **1**), the glucosyl moiety changed its bonding position from 7-*O* position of the aglycone to C-5 position of the β-apiosyl residue (see Table 3). The compound was therefore identified as isorhamnetin-3-*O*-[β-d-glucopyranosyl-(1→5)-β-d-apiofuranosyl]-(1→2)-robinobioside or [α-l-rhamnopyranosyl-(1→6)]-β-d-galactopyranosyl], a newly described flavonoid glycoside.

Compound **3** was isolated as a yellow amorphous powder. Rt of compound **3** was 4.61 min, again slightly different from other isolates. The HR-ESI-MS in a negative mode displayed a molecular ion *m/z* [M − H]^−^ 931.27352 (calcd [M − H]^−^ 931.272491), confirming molecular formula C_40_H_52_O_25_ with calculated molecular mass 932.82588 Da. HRESIMS showed additional adduct formation at *m/z* [M + HCOO]^−^ 977.27862, and a hexose loss (*m/z* 162.05387) leading to *m/z* 769.21965 (representing supposed cleavage of glucose from 7-*O* position). In the HRAM ESI positive mode, [M + H]^+^ ion with *m/z* 933.2810 (calcd [M + H]^+^ 933.2876) was obtained. Fragmentation with collision energy of 20 eV gave ions with *m/z* 787.2229 and *m/z* 771.2324 formed by loss of hexose and rhamnose, respectively. Further subsequent loses of the hexosyl and rhamnosyl moieties resulted fragments with *m/z* 625.1679 (aglycone-rhamnosyl-hexoside) and *m/z* 641.1652 (aglycone-hexosyl-hexoside). Finally, ions of *m/z* 479.1082 (hexosylated aglycone) and aglycone *m/z* 317.0526 (supposed isorhamnetin) were found. Together with an interpretation of NMR spectra (Table 2 and Table 3), the structure was predicted to be composed of isorhamentin and four sugar moieties. Two of them were recognized as 6-deoxyhexoses (two methyl groups in the form of the doublet), and the other two were hexoses (possibly a glucose, or a galactose). DEPT spectrum showed their two methylene groups (δ_C_ 61.1 ppm and 65.9 ppm) that belonged to the galactose and the glucose, respectively. We observed four anomeric protons in HSQC spectrum: δ_H_ 5.79 ppm, (1H, d, *J* = 7.84 Hz), δ_H_ 5.16 ppm (1H, d, *J* = 1.52 Hz), δ_H_ 4.53 ppm (1H, d, *J* = 1.53 Hz) and δ_H_ 5.06 ppm (1H, d, *J* = 7.24 Hz), corresponding to carbon atoms δ_C_ 99.3 ppm, 101.4 ppm, 100.6 ppm and 100.1 ppm, respectively. After complete resonance assignments and analyses of coupling constants, intensities of cross-peaks in the COSY spectrum, and ^13^C-NMR chemical shift values, one hexose moiety was identified as a β-glucosyl unit, the other as a β-galactosyl moiety, and the 6-deoxy sugars were found to be α-rhamnosyl moieties. ^13^C values indicated that β-glucosyl residue is free at C-6 (δ_C_ 61.1 ppm), while the β-galactose is bonded to C-6 (δ_C_ 65.9 ppm—shifted to a higher field). According to HMBC correlations, structure of the side chains and their attachment to the aglycone were established. Anomeric proton (δ_H_ 5.06 ppm) of the glucose moiety showed a three-bond correlation to C-7 (δ_C_ 163.0 ppm) of the aglycone, while an HMBC correlation between the anomeric proton of the *β*-galactosyl moiety (δ_H_ 5.79 ppm) and the carbon at δ_C_ 133.3 ppm indicated that the *β*-galactosyl unit is bonded at C-3 toward the aglycone. The anomeric proton of one of the *α*-rhamnose residues (δ_H_ 5.16 ppm) was correlated to position C-2 of the β-galactose (δ_C_ 76.4 ppm), while the anomeric proton of the other α-rhamnose showed correlation to C-6 of the β-galactose (δ_C_ 65.9 ppm). Methyl residues of α-rhamnopyranosyl residues were located in the low-field region of ^1^H spectrum at δ_H_ 0.89 ppm, (3H, d, *J* = 6.25 Hz) for the α-rhamnosyl residue attached to C-2 of the β-galactose and at δ_H_ 1.16 ppm (3H, d, *J* = 6.08 Hz) for the α-rhamnosyl residue attached to C-6 of the β-galactose, respectively. Hence, we identified compound **3** as the new natural product isorhamnetin-3-*O*-(2,6-di-*O*-α-rhamnopyranosyl-β-d-galactopyranoside)-7-*O*-β-d-glucopyranoside. The compound is similar to compound **7**; the difference lies in the absence of β-d-glucopyranosyl moiety at 7-*O* position in compound **7**.

Compound **4** was isolated as a yellow amorphous powder. Chromatographic analysis showed Rt 5.40 min. The HR-ESI-MS in negative mode displayed a molecular ion *m/z* [M − H]^−^ 785.21509 (calcd for [M − H]^−^ 785.21403), confirming molecular formula C_34_H_42_O_21_ with calculated molecular mass 786.68468 Da. HRESIMS in negative mode showed additional adduct formation *m/z* [M + HCOO]^−^ 831.22089, and a hexose (*m/z* 162.05404) loss leading to *m/z* 623.16174 (representing cleavage of glucose from 7-*O* position or a galactose from 3-*O*). The moderate fragmentation in positive ESI (20 eV) showed the presence of *m/z* 787.2284 for parental ion, and then the corresponding fragment with a cleavage of deoxyhexose *m/z* 641.1714, hexose *m/z* 625.1753, two sugar (rhamnosyl and hexosyl) units *m/z* 479.1178 and an aglycone at *m/z* 317.0658, possible isorhamnetin. After the interpretation of NMR spectra (Table 2 and Table 3), we confirmed the presence of isorhamnetin and three sugar moieties. Compound **4** possesses similar structure as compound **3**, with absence of the *α*-rhamnopyranosyl moiety connected to C-2 of β-galactosyl residue, and we identified compound **4** as isorhamnetin-3-*O*-robinobioside-7-*O*-glucoside, a new flavonoid glycoside.

## 3. Discussion

As described above, we isolated 7 flavonoid compounds from *A. aitosensis* extract. Their structures were elucidated by 1D (^1^H, ^13^C) and 2D NMR experiments (COSY, HSQC, HMBC, NOESY and TOCSY) and confirmed by HR-ESI-MS. We report the structures of six isorhamnetin and one kaempferol glycosides (**1**−**7**), including three new tetra- (**1**–**3**), one new tri-glycoside (**4**) and three already known compounds with two (**5**), three (**6**) and four (**7**) sugar units, respectively.

Genus *Astragalus* is one of the largest genera of Fabaceae family. As mentioned, bioactivity of *Astragalus* plants are connected with a presence of flavonoids, saponins and polysaccharides. The use of *Astragalus* spp. is mainly connected with immunomodulation, antibacterial and antiviral activity, and hepatoprotection [9,10]. The reviews of Gorai et al. [16], Bratkov et al. [10], and Li et al. [17] show an overview of *Astragalus* genera and flavonoids isolated, showing the presence of flavones, flavonols, flavanones, flavan-4-ols, isoflavones, isoflavans, pterocarpans and others in 60 different *Astragalus* species. Their reviews also include isorhamnetin and kaempferol glycosides. Another comprehensive review of Bulgarian *Astragalus* species, published in 2016 [18], similarly describes the presence of several kaempferol and isorhamnetin glycosides, including alangiflavoside from *A.* *monspessulanus* ssp. *monspessulanus* [18,19].

In recent years, Bulgarian researchers isolated and reported new tri- and tetraglycosides of flavonols, including some new compounds from the rarely-met group of flavo-alkaloids [10,11]. Many species of *Astragalus* possess in nature the widely-distributed aglycones—kaempferol, quercetin and methylquercetines—in their free and glycosidic forms [10]. *A. aitosensis* has previously shown presence of rutin, quercetin-3-*O*-β-d-glucoside and astragalin [7]. As visible from comparison of glycosides isolated from *A. aitosensis* with the literature, similar compounds—glycosides—were obtained for example from A. *monspessulanus* ssp. *monspessulanus, A. cicer* and A. centralpinus [18,19,20]. This may, from chemotaxonomic point of view, confirm their close relationships.

## 4. Materials and Methods

### 4.1. General Experimental Procedures

Optical rotations were measured on a JASCO P-2000 spectropolarimeter (Easton, MD, USA) at 20 °C in MeOH with Spectramanager software.

HPLC used configuration of analytical system by Agilent 1100 Series (Degasser G1322A, Quaternary Pump G1311A, Autosampler ALS G1313A, Column Compartment G1316, DAD G1315B, Loop 20 µL, UV spectrum 200–900 nm) with column Kinetex^®^ PFP 100 A, 250 mm × 4.6 mm I.D., 5 µm (Phenomenex, CA, USA), and flow rate of 1 mL/min. Semi-preparative HPLC was carried out using Dionex UltiMate 3000 system (Pump Dionex UltiMate 3000 UPLC+ Focused, Dionex UltiMate 3000 RS Variable Wavelength Detector, fraction collector Dionex UltiMate 3000 with 6 positions, LCO 101 ECOM column oven, constant temperature 40 °C, autosampler Dionex UltiMate 3000, loop 100 µL), column Ascentis^®^ RP-AMIDE, 250 mm × 10 mm, 5 µm (Supelco, PA, USA), and flow rate of 5 mL/min. TLC was carried out on precoated silica gel plates (Supelco Kieselgel G, F254, 60, Merck, Darmstadt, Germany) with the solvent systems EtOAc:MeOH:H_2_O (100:13.5:10, *v*/*v*/*v*). Spots were visualized under UV light (365 nm) after spraying with NTS/PEG reagent. Column chromatography (CC) was performed using Diaion HP-20 (Supelco, PA, USA), Ø = 80 mm, height 70 cm ~ 700 g and Silica gel (40−63 μm, Sigma-Aldrich^®^, St. Louis, MO, USA) Ø = 35 mm, height 60 cm.

### 4.2. Plant Material

The aerial parts of *A. aitosensis* (Ivan.) Podl (Fabaceae) (syn. *Astracantha aitosensis* (Ivan.) Podl.) was collected and identified by Hristo Vasilev in June 2015 in the suburbs of town Aytos, Bulgaria (coordinates Google maps: 42.702191 N, 27.266976 E, UTM: NH22), voucher specimen has been deposited in Herbarium of the Institute of Biodiversity and Ecosystem Research, Bulgarian Academy of Sciences with Ref No. SOM001362.

### 4.3. Extraction and Isolation

Dried aerial parts (3600 g) of *A. aitosensis* were extracted under reflux with 80% MeOH (20 × 1.25 L, 40 min each) at 70 °C. The total methanol extract was evaporated to dryness (128 g, 3.5%), and it was re-suspended in H_2_O (800 mL) to remove nonpolar compounds by liquid–liquid extraction with chloroform (200 mL × 5), giving 18 g of CHCl_3_ fraction and 110 g of MeOH soluble material. This defatted methanol portion was subjected to Diaion HP-20 column, and eluted with gradient system of H_2_O and MeOH (100:0 to 0:100, *v*/*v*), giving 10 combined fractions assigned A–J. Fraction B, C, E, and F were further purified via open column chromatography (Silica gel, eluted with CHCl_3_:MeOH:H_2_O 6:4:0.2, *v*/*v*/*v*). Fraction B was re-chromatographed on silica gel (CHCl_3_:MeOH:H_2_O 6:4:0.2, *v*/*v*/*v*, as mobile phase,) and resulted in 6 combined fractions (B_1_–B_6_). Fraction B_4_ was further purified using a semipreparative HPLC (gradient of acetonitrile in 0.2% HCOOH from 12 to 20% in 25th minute), and this purification gave compound **4** (27 mg). Fraction B_5_, after semipreparative HPLC (gradient of acetonitrile in 0.2% HCOOH from 15% to 18% in 20th minute), gave compound **1** (11 mg). Fraction B_6_ was purified on semi-preparative HPLC (gradient of acetonitrile in 0.2% HCOOH from 15% to 20% in 20th minute), gave compounds **7** (20 mg) and **3** (45 mg), respectively. Fraction C was re-chromatographed on silica gel (mobile phase 6:4:0.2 CHCl_3_:MeOH:H_2_O, *v*/*v*/*v*) and this separation resulted in 45 fractions. Pure compound **2** (24 mg) precipitated from fraction C_34_. Three hundred milligrams of fraction E were purified with semipreparative HPLC (gradient of acetonitrile in 0.2% HCOOH from 19 to 22% in 18th minute), and gave compound **6** (56 mg). Fraction F was re-chromatographed on silica gel (mobile phase 6:4:0.2 CHCl_3_:MeOH:H_2_O, *v*/*v*/*v*), which resulted in 35 fractions, 100 mL each. Pure compound **5** (40 mg) yielded from fraction F_10_ after re-crystallization in MeOH.

### 4.4. Identification of Isolated Compounds

NMR spectra were recorded on a NMR Agilent DD2 600 MHz (compounds **1**–**7**) and a NMR Agilent VNMRS 500 MHz (compound **2**)—equipped with four and three channels, respectively, and structure elucidation was carried out using modern 1D and 2D pulse sequences, following 1D and 2D experiments were carried out: ^1^H, ^13^C, COSY, HMBC, HSQC, TOCSY, and NOESY. The spectra were processed with MestReNova version 12.0.0 (Mestrelab Research, Santiago de Compostela, Spain). Mass spectra were recorded using a Thermo Scientific Q Exactive Plus quadrupole—Orbitrap mass spectrometer coupled with a UPLC Dionex Ultimate 3000 RSLC system equipped with an RP-18 Kinetex column (2.10 mm × 100 mm, 2.6 μm, Phenomenex Corporation, Torrence, CA, USA). Full-scan data were recorded in negative ESI mode from *m/z* 100 to 1500 at a resolution of 70,000 (at *m/z* 200). Full-scan dd-MS^2^ (Top 5) was performed at a resolution of 17 500 (at *m/z* 200), AGC target 1^e5^ with maximum IT 30 ms. For HR-MS in positive mode, Q-TOF mass spectrometer with ultra-high resolution and high mass accuracy (HRAM) Impact II (Bruker Daltonik, Bremen, Germany) were used. UHPLC Dionex UltiMate 3000 (Thermo Fisher Scientific, Waltham, MA, USA) was used for LC, with mobile phases: 0.1% formic acid (A) and MeOH (B), flow rate: 0.2 mL/min, gradient elution: 0 min 5% of B, 3.6 min 10% of B, 10th min 100% of B. Column block temperature was 35 °C, and injection volume 5 µL. Kinetex P5, 100A, 1.7um, 100 × 2.1 mm (Phenomenex, Torrance, CA, USA) was used as column. Solution of natrium formate clusters was used as calibration mixture for accurate mass calibration, with MS source settings: end plate offset: 500 V, capillary 4500 V, nebulizer pressure 0.3 Bar, dry gas: 4.0 L/min, dry temperature: 250 °C. MS/MS spectra were collected at three collision energy levels of 20, 40, and 60 eV per each peak. Data acquisition were carried out by Control 4.0 and HyStar 3.2 software and the results were processed using Compass DataAnalysis 4.3 (all SW of Bruker Daltonik, Bremen, Germany). Chromatography was controlled by Chromeleon Xpress link (Thermo Fisher Scientific, Waltham, MA, USA). For fragmentation analysis Mass Frontier 7.0.5.9 SR3 (High Chem Ltd., Bratislava, Slovakia) and Thermo Excalibur 3.0.63 (Thermo Fisher Scientific, Waltham, MA, USA) software was used.

## Figures and Tables

**Table 1:** ^13^C-NMR δ_C_ (ppm) (100 MHz); ^1^H-NMR (600 MHz for **1** and 500 MHz for **2**). δ_H_ (ppm), multiplicity (*J* in Hz).

	1		2	
	δ_C_, Type	δ_H_ (*J* in Hz)	δ_C_, Type	δ_H_ (*J* in Hz)
**2**	157.8, C		157.2, C	
**3**	133.6, C		133.3, C	
**4**	178.0, C		177.8, C	
**5**	161.3, C		161.6, C	
**6**	99.4, CH	6.45, d (2.02)	98.5, CH	6.19, d (2.00)
**7**	163.0, C		164.4, C	
**8**	94.4, CH	6.75, d (2.02)	93.4, CH	6.38, d (2.00)
**9**	156.5, C		157.0, C	
**10**	106.3, C		104.5, C	
**1′**	121.8, C		121.9, C	
**2′**	113.3, CH	8.05, m (1.94)	113.7, CH	8.04, d (1.97)
**3′**	147.0, C	-OCH_3_	147.0, C	-OCH_3_
**4′**	149.4, C	-OH	149.2, C	-OH
**5′**	114.5, CH	6.91, m (8.39)	114.5, CH	6.90, d (8.40)
**6′**	122.3, CH	7.62, m (1.92; 8.45)	122.1, CH	7.57, dd (1.90; 8.43)
**-*O*CH_3_**	55.7, CH	4.00 s	55.7, CH_3_	3.98, s
**3-*O*-gal**				
**1**	99.8, CH	5.57, d (7.84)	99.9, CH	5.58, d (7.76)
**2**	75.1, CH	3.96, dd	74.8, CH	3.97, dd
**3**	73.9, CH	3.73 dd	73.9, CH	3.74 dd
**4**	69.0, CH	3.77 dd	69.0, CH	3.77 dd
**5**	74.1, CH	3.67 dt	74.0, CH	3.64 dt
**6**	66.0, CH_2_	3.47/3.68 dd	65.8, CH_2_	3.47/3.71 dd
**Api (1→2)**				
**1**	109.2, CH	5.43, d (1.55)	109.1, CH	5.44, d (1.73)
**2**	76.6, CH	4.01, d	76.9, CH	4.07, d
**3**	79.5, C	-OH	78.8, C	-OH
**4**	74.2, CH_2_	3.64, d (9.58)	74.3, CH_2_	3.66, d (n/a)
		3.75, d (9.59)		4.07, d (9.70)
**5**	65.1, CH_2_	3.64, d (11.45)	61.3, CH_2_	3.67, d (11.95)
		3.75 d (11.56)		3.85 d (11.90)
**Rha (1→6)**				
**1**	100.5, CH	4.50, d (1.56)	100.5, CH	4.52, d (1.53)
**2**	70.7, CH	3.51, dd	70.7, CH	3.57, dd
**3**	70.9, CH	3.46, dd	70.9, CH	3.49, dd
**4**	72.4, CH	3.25, pt	72.4, CH	3.26, pt
**5**	68.3, CH	3.49, dq	68.3, CH	3.51, dq
**6**	16.5, CH_3_	1.15, d (6.17)	16.5, CH_3_	1.16, d (6.23)
**Glc**	**7-O-Glc**		**Api (5→1) Glc**	
**1**	100.1, CH	5.06, d (7.53)	103.4, CH	4.25, d (7.54)
**2**	73.3, CH	3.49, dd	73.6, CH	3.17, dd
**3**	77.0, CH	3.55, dd	76.3, CH	3.21, dd
**4**	69.9, CH	3.38, dt	70.4, CH	3.27, dd
**5**	76.4, CH	3.49, dq	76.3, CH	3.27, dt
**6**	61.1, CH_2_	3.69/3.94 dd	72.9, CH_2_	3.68/4.15, dd

**Table 2:** ^13^C-NMR assignments (100 MHz) for compounds **3**–**7**, δ (ppm).

Compound/Position	3	4	5	6	7
	δ_C_, Type	δ_C_, Type	δ_C_, Type	δ_C_, Type	δ_C_, Type
**2**	157.7, C	157.4, C	156.8, C	157.0, C	158.0, C
**3**	133.3, C	133.8, C	133.5, C	133.0, C	133.4, C
**4**	177.9, C	178.0, C	177.8, C	177.8, C	177.1, C
**5**	161.3, C	161.3, C	161.6, C	161.7, C	161.4, C
**6**	99.3, CH	99.8, CH	99.2, CH	98.4, CH	99.4, CH
**7**	163.0, C	163.4, C	164.7, C	164.4, C	163.0, C
**8**	94.4, CH	95.1, CH	94.2, CH	93.3, CH	94.3, CH
**9**	156.5, C	156.4, C	156.8, C	157.0, C	156.6, C
**10**	106.3, C	106.1, C	104.4, C	104.5, C	106.2, C
**1′**	121.8, C	121.3, C	121.5, C	122.0, C	121.5, C
**2′**	113.3, CH	113.9, CH	113.9, CH	113.3, CH	131.0, CH
**3′**	147.1, CH	147.5, CH	147.4, C	147.0, C	114.8, CH
**4′**	149.3, C	150.1, C	149.9, C	149.1, C	160.1, C
**5′**	114.6, CH	115.6, CH	114.6, CH	114.5, CH	114.8, CH
**6′**	122.1, CH	122.6, CH	122.4, CH	121.8, CH	131.0, CH
***O*CH_3_**	56.37, CH_3_	56.39, CH	56.37, CH	55.79, CH_3_	
**3-*O*-Gal**					
**1**	99.3, CH	102.1, CH	102.3, CH	99.4, CH	99.4, CH
**2**	76.4, CH	74.1, CH	74.0, CH	76.4, CH	76.1, CH
**3**	74.2, CH	71.6, CH	71.5, CH	74.2, CH	74.3, CH
**4**	69.2, CH	68.4, CH	68.4, CH	69.1, CH	69.4, CH
**5**	74.1, CH	73.4, CH	73.4, CH	73.9, CH	74.1, CH
**6**	65.9, CH_2_	65.6, CH_2_	65.6, CH_2_	65.7, CH_2_	66.0, CH_2_
**Rha (1→2)**					
**1**	101.4, CH			101.3, CH	101.2, CH
**2**	71.0, CH			71.0, CH	71.0, CH
**3**	71.0, CH			71.0, CH	70.9, CH
**4**	72.5, CH			72.5, CH	72.7, CH
**5**	68.4, CH			68.4, CH	68.4, CH
**6**	16.0, CH_3_			16.0, CH_3_	16.1, CH_3_
**Rha (1→6)**					
**1**	100.6, CH	100.5, CH	100.5, CH	100.5, CH	100.5, CH
**2**	70.7, CH	70.9, CH	70.9, CH	70.7, CH	70.7, CH
**3**	70.9, CH	71.1, CH	71.1, CH	70.9, CH	70.9, CH
**4**	72.4, CH	72.2, CH	72.3, CH	72.6, CH	72.5, CH
**5**	68.3, CH	68.7, CH	68.7, CH	68.3, CH	68.3, CH
**6**	16.6, CH_3_	18.3, CH_3_	18.3, CH_3_	16.6, CH_3_	16.6, CH_3_
**7-*O*-Glc**					
**1**	100.1, CH	100.3, CH			100.1, CH
**2**	73.3, CH	73.6, CH			73.3, CH
**3**	77.0, CH	76.9, CH			77.0, CH
**4**	69.9, CH	70.0, CH			69.9, CH
**5**	76.4, CH	77.7, CH			76.4, CH
**6**	61.1, CH_2_	61.2, CH_2_			61.1, CH_2_

**Table 3:** ^1^H-NMR assignments (600 MHz) of compounds **3**–**7** δH (ppm), multiplicity (*J* in Hz).

	3	4	5	6	7
	δ_H_ (*J* in Hz)	δ_H_ (*J* in Hz)	δ_H_ (*J* in Hz)	δ_H_ (*J* in Hz)	δ_H_ (*J* in Hz)
**2**					
**3**					
**4**					
**5**					
**6**	6.44, d (2.07)	6.44, d (1.91)	6.19, d (1.97)	6.15, d (1.97)	6.46, d (2.15)
**7**					
**8**	6.75, d (2.09)	6.77, d (1.97)	6.42, d (1.97)	6.37, d (1.97)	6.75, d (2.14)
**9**					
**10**					
**1′**					
**2′**	8.09, m (1.93)	8.00, d (1.84)	7.98, d (1.97)	8.07, d (1.94)	8.09, m (8.95)
**3′**					6.90, m (8.90)
**4′**					
**5′**	6.91, m (8.44)	6.90, d (8.39)	6.88, d (8.44)	6.90, d (8.42)	6.90, m (8.90)
**6′**	7.57, m (1.91; 8.42)	7.54, dd (1.82; 8.48)	7.49, dd (2.03; 8.39)	7.52, dd (1.99; 8.43)	8.09, m (8.95)
***O*CH_3_**	3.83, s	3.84, s	3.83, s	4.00, s	-
**3-*O*-Gal**					
**1**	5.79, d (7.84)	5.47, d (7.73)	5.45, d (7.67)	5.59, d (7.83)	5.59, d (7.74)
**2**	3.97, dd	3.60, dd	3.57, dd	3.96, dd	3.95, dd
**3**	3.78, dd	3.57, dd	3.57, dd	3.76, dd	3.70, dd
**4**	3.80, dd	3.62, dd	3.63, dd	3.81, dd	3.75, dd
**5**	3.74, dt	3.42, dt	3.41, dt	3.71, dt	3.64, dt
**6**	3.54/3.73dd	3.30/3.60 dd	3.31/3.61 dd	3.52/3.72 dd	3.46/3.69 dd
**Rha (1→2)**					
**1**	5.16, d (1.52)			5.16, d (1.53)	5.21, d (1.32)
**2**	4.00, dd			4.00, dd	4.00, dd
**3**	3.75, dd			3.76, dd	3.78, dd
**4**	3.33, pt			3.32, pt	3.33, pt
**5**	4.04, dq			4.03, dq	4.06, dq
**6**	0.89, d (6.25)			0.89, d (6.23)	0.98, d (6.23)
**Rha (1→6)**					
**1**	4.53, d (1.53)	4.41, d (1.53)	4.41, d (1.53)	4.55, d (1.55)	4.49, d (1.24)
**2**	3.54, dd	3.37, dd	3.38, dd	3.59, dd	3.48, dd
**3**	3.49, dd	3.28, dd	3.28, dd	3.51, dd	3.46, dd
**4**	3.25, pt	3.06, pt	3.07, pt	3.26, pt	3.25, pt
**5**	3.52, dq	3.36, dq	3.34, dq	3.54, dq	3.49, dq
**6**	1.16, d (6.08)	1.04, d (6.18)	1.03, d (6.25)	1.17, d (6.22)	1.16, d (6.21)
**7-*O*-Glc**					
**1**	5.06, d (7.24)	5.05, d (7.89)			5.07, d (7.45)
**2**	3.48, dd	3.24, dd			3.48, dd
**3**	3.53, dd	3.27, dd			3.53, dd
**4**	3.39, dd	3.15, dd			3.38, dd
**5**	3.50, dt	3.43, dt			3.49, dt
**6**	3.69/3.92, dd	3.44/3.68, dd			3.69/3.91, dd

## Supplementary Materials

The following are available online. Figures S1–S77: NMR spectra of compounds **1**–**7**, HRMS of compounds **1**–**7**, and HPLC chromatograms of compounds **1**–**7**.
